# Automated Virtual Fencing Can Effectively Contain Sheep: Field Trials and Prospects

**DOI:** 10.3390/ani13040619

**Published:** 2023-02-10

**Authors:** Dana L. M. Campbell, Sue Belson, Jim M. Lea, Jackie Ouzman, Caroline Lee, Troy Kalinowski, Damian Mowat, Rick S. Llewellyn

**Affiliations:** 1 Agriculture and Food, Commonwealth Scientific and Industrial Research Organisation (CSIRO), Armidale, NSW 2350, Australia; 2 Agriculture and Food, Commonwealth Scientific and Industrial Research Organisation (CSIRO), Glen Osmond, SA 5064, Australia

**Keywords:** GPS, behaviour, sheep, electrical pulse, audio cue, grazing

## Abstract

**Simple Summary:**

Virtual fencing technology uses on-animal devices to communicate boundaries via a warning audio tone and electrical pulse signals. There are limited virtual fencing studies on sheep. This study used modified cattle eShepherd^®^ virtual fencing neckbands to enable automated trials with small sheep groups. The first 5-day trial with six Dorper crossbred sheep was conducted in an experimental paddock setting, with a second 5-day trial conducted with 10 Ultra White sheep on a commercial farm. The animals across both trials were contained in the inclusion zone for 99.8% and 92.2% of the trial period, and most of the cues they received were audio tones, indicating they were learning the warning audio tone meant to stop or turn around. In the second trial, sheep crossed over into the exclusion zone on the third night and remained there until they were walked out for their daily yard check in the morning. These preliminary trial results indicate automated technology can work on sheep, but devices need to be designed specifically for sheep, including algorithms adapted to better herd a group of sheep back out of an exclusion zone. A collar device may only be applicable to some sheep breeds with reduced wool.

**Abstract:**

Virtual fencing technology uses on-animal devices to communicate boundaries via a warning audio tone and electrical pulse signals. There is currently limited validation work on sheep. This study used modified cattle eShepherd^®^ virtual fencing neckbands on reduced-wool sheep with clipped necks to enable automated trials with small groups across both day and night. The first 5-day trial with six Dorper crossbred sheep was conducted in an experimental paddock setting, with a second 5-day trial conducted with 10 Ultra White sheep on a commercial farm. The animals across both trials were contained in the inclusion zone for 99.8% and 92.2% of the trial period, with a mean percentage (±SD) of total audio cues as audio only (i.e., not followed by an electrical pulse) being 74.9% ± 4.6 in the first trial, and 83.3% ± 20.6 for the second trial. In the second trial, sheep crossed over into the exclusion zone on the third night and remained there until they were walked out for their daily yard check in the morning. These preliminary trial results are promising for the use of automated technology on sheep, but suitable devices and algorithms still need to be designed specifically for sheep in the long term.

## 1. Introduction

Virtual fencing represents an emerging technology that has the potential to fence and move livestock for enhanced grazing control and monitoring. Virtual fencing communicates the presence of boundaries to animals through audio and electrical pulse signals administered to individuals via a device worn by the animal, such as a neckband. The animals are trained to associate the audio warning tone with the negative stimulus to avoid virtual boundaries based on sound only [1,2,3,4]. There are several types of virtual fencing neckband devices that are currently being commercialised for use on beef and dairy cattle [1,2,3,4,5], goats [6], and sheep [7,8]. While there has been demonstrated success of the technology with cattle across a variety of paddock situations and different device designs [1,2,3,9,10,11], to date there has been limited success in applying automated technology to sheep. The fleece of sheep presents a barrier to successful administration of electrical pulses relative to cattle, and their smaller body weight places greater restrictions on device design. The automated Nofence devices (Nofence, AS, Batnfjordsøra, Norway) have been tested on sheep [7,8]. These devices used a 4s audio tone of increasing frequency when an animal reached the virtual border, followed by an electric shock if they kept moving forward [7,8]. However, the authors concluded the technology was a significant welfare concern due to high individual variation in reactivity to the electric shocks and learning rates that resulted in sheep not meeting predetermined learning criteria [7,8]. These results may have, in part, been due to the stage of the technology development at the time of the trials [7,8]. Earlier trials with dog collars and a fixed buried wire showed ewes did learn the concept of the virtual fencing cues after only a few experiences with the ‘line’, but the buried wire approach is limited in its commercial application [12]. Audio-only devices have also been tested, where there is no electrical pulse, and aversive audio cues are administered [13]. On average, 10% of animals across a small test group showed no response to the different audio stimuli tested, which may limit the system’s application [13].

In the absence of suitable automated virtual fencing devices for sheep, manual electronic dog collars applying audio and electrical stimuli in a similar manner to the eShepherd^®^ automated cattle devices have been used across a range of studies to assess the learning capabilities of sheep, their responses to the stimuli, and potential commercial applications of the technology [14,15,16,17,18,19]. These manual trials have relied on visual observation by trial operators to apply a one second audio cue followed by an up to one second electrical pulse if the sheep kept moving forward, and as such have been limited to short-term trials during daylight hours only. When tested individually, sheep learned the association between the audio cue and the electrical pulse and exhibited the correct behaviour of stopping and/or turning around upon hearing the audio cue alone [14,15]. High individual variation in the rate of learning was reported, but all sheep during individual testing were able to learn the audio/electrical pulse association [14,15]. Sheep in a small group in a commercial paddock setting were excluded from a specific area and rapidly returned to the previously excluded area once the fence cues were no longer applied, indicating that they were learning to respond to the audio cue and not the location [16]. A virtual fence was also comparable to an electric fence for targeted grazing within small plots and did not result in negative changes in grazing behaviour [17]. Interestingly, there was evidence in the small (nine animal) short-term trial scenario that not all sheep were required to wear neckbands. Virtual fencing was effective when 66% of sheep were wearing devices, indicating social effects on responses to the fence cues; however, the fence was not effective when only 33% of animals were collared [18]. Finally, a back fence was able to be implemented to prevent movement back over previously grazed areas [19]. These studies all indicate the potential for virtual fencing technology to be applied to sheep, but any larger studies, including ones that run across the day and the night, are limited by the logistics of requiring personnel to manually operate the individual devices throughout the study period.

The eShepherd^®^ virtual fencing devices are currently being commercialised for predominantly beef cattle by Gallagher (Hamilton, New Zealand). The pre-commercial system comprises neckband devices, a base station on site to communicate with the devices, and an online user interface for animal and fence management. Previous research on beef cattle using varying iterations of the pre-commercial eShepherd^®^ prototypes have demonstrated that all tested cattle will learn to respond to the audio cue alone to avoid receiving electrical pulses, but with individual variation in learning rate and frequency of fence interactions [9,10,11]. Beef cattle have been excluded from environmentally sensitive areas such as riparian zones [10] and regenerating sapling plantings [11] and have been comparably excluded from areas of pasture when using virtual fences or electric tape fences [9]. There is potential for a modified version of this device to be applied to sheep to enable automated research trials.

The aim of this study was to apply modified eShepherd^®^ virtual fencing neckbands to sheep to enable automated trials with small sheep groups across both day and night. These were the first trials conducted using these specific automated GPS-based devices on sheep. It was predicted that sheep would learn the association between the audio cue and electrical pulse and would remain within the inclusion zone.

## 2. Materials and Methods

### 2.1. eShepherd^®^ Neckbands

The virtual fencing pre-commercial prototype (eShepherd^®^, Gallagher, Hamilton, New Zealand) system was used in these trials and has been previously described [9,10,11]. The cattle neckband consisted of a strap and hanging counterweight (total weight approximately 1.4 kg) and a unit (approximately 730 g and 17 cm L × 12 cm W × 13 cm H), positioned on top of the neck. However, this was not suitable for sheep due to their smaller size, and thus the device was modified to fit the unit onto the sheep’s back via a harness, as described in Section 2.2 *Chiswick pilot trial—animals and experimental protocol*. The unit used GPS technology to monitor animal movement and provided a real-time measure of animal position, heading, and speed. A virtual fence boundary (delineating inclusion and exclusion zones) was specified using GPS coordinates and transmitted to the unit via a radio frequency link from a base station installed adjacent to the test paddock. As an animal approached within 5 m of the virtual fence boundary, the unit emitted a non-aversive audio tone. If the animal stood still or turned away, no electrical pulse was applied. If the animal continued to move through the virtual fence boundary into the exclusion zone, the unit delivered a short, sharp electrical pulse sequence in the kilovolt range (values were known to researchers but are commercial in confidence). This audio-pulse sequence was repeated if the animal continued to walk through the fence line into the exclusion zone. If the animal turned around and moved back toward the inclusion zone, all stimuli ceased. If animal movement occurred above or below a specified velocity (values are commercial in confidence), stimuli were not applied. If an individual animal received a specified number of stimuli within a specified time frame, the device entered standby mode, and stimuli were not applied for a specified time frame (values were known to the researchers but are commercial in confidence). An online user interface allowed real-time monitoring of animal movement. All neckband cues (audio and electrical pulse stimuli) and GPS locations were downloaded from cloud storage.

### 2.2. Chiswick Pilot Trial—Animals and Experimental Protocol

**Figure 1:**
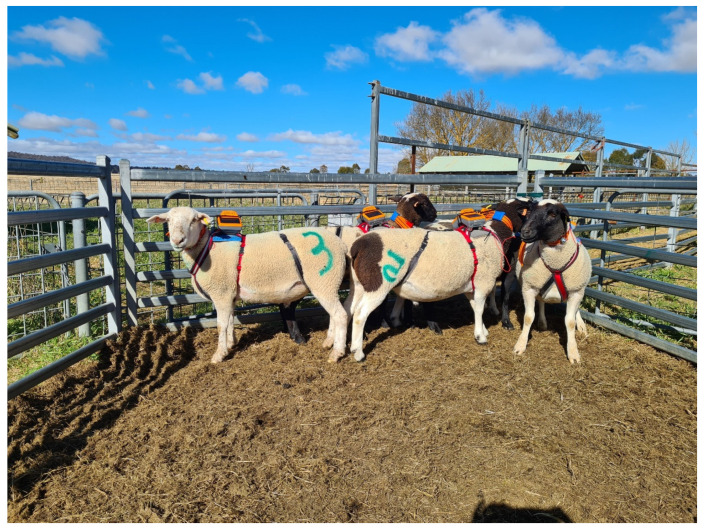
Gallagher eShepherd^®^ units fitted to the sheep using a modified dog harness including the wired collar around the neck containing the electrodes. The fit of each harness was checked to ensure normal movement by the animals and no obvious signs of discomfort resulting from the straps.

All testing in the pilot trial occurred at the CSIRO Chiswick Research Station in Armidale, NSW, in May–July 2022. Across the pilot trial, a total of 37 Dorper crossbred wethers (12–24 months of age), naïve to virtual fencing, with an average weight of 57.3 (±0.80) kg were used. The Dorper sheep are a non-wool sheep breed that is becoming increasingly common in Australian crop-livestock farming systems [20] and they were selected for this trial due to their hairier coats being less likely to insulate and block the electrical pulse. The cattle eShepherd^®^ device was planned to be used on sheep, as it is a functional automated device validated across many previous cattle research trials (e.g., [9,10,11]), and there is no other available automated device within Australia that has been demonstrated to be suitable for sheep. However, the device was designed for cattle, and thus it was unclear how it may be applied to sheep, with smaller body size and different coats. A series of preliminary tests occurred to determine how to fit the unit to sheep and the electrical pulse level that would be aversive to the sheep without causing adverse behavioural responses. Through trial and error of fit and nine short tests with small numbers of sheep (up to 6/group) a custom-modified dog harness was eventually created that fit the eShepherd^®^ unit onto the backs of sheep with wires that connected the unit to electrodes placed on a collar around the animal’s neck (Figure 1). Necks were clipped to ensure consistency of any insulative cover. This design was selected, as the unit was able to remain upright on the sheep’s back. The electrical pulses were applied to the front of the neck as per previous trials with sheep using manual collars (e.g., [14,16]); sheep movement was not observed to be restricted by the harness. The electrical pulse level was reduced relative to what is applied to cattle (exact values were known to the researchers but are commercial in confidence). The electrical pulse level initiated similar behavioural reactions to those reported in previous studies [14,21].

Once the fit of the unit and a suitable electrical pulse level were selected, a group of six sheep that had not previously been used for any audio tone and electrical pulse testing were restrained in a race and fitted with the harness devices. The neck was clipped, and a coloured wool marker (Heiniger Shearing Supplies, Bibra Lake, WA, Australia) was used to number each sheep. These animals had been used for testing different harness/collar designs a few weeks prior (no stimuli applied) and thus were partially accustomed to wearing the devices. They were allowed 30 min in the yards to habituate further to the devices following fitting of the last animal before being placed into a small test paddock (0.61 ha) adjacent to the yards on day one. Animals that were first fitted had longer to habituate, as the process of fitting took approximately 45 min. The animals were observed to settle within a few minutes or less of being fitted with the devices but were still given a minimum of 30 min to ensure acclimation. The test paddock had plentiful grazing feed available, with a mix of native and introduced pasture and a single water point within the inclusion zone. Animals were walked through the test paddock to the top and held there by personnel standing in front of the group while a single virtual fence line was set across the width of the paddock to exclude 25% of the paddock area (exclusion zone). Following fence activation, personnel left the paddock, and animals were observed from a neighbouring paddock. This was to confirm the sheep were able to learn the audio and electrical pulse stimuli association during their first interactions with a virtual fence line by starting to respond to the audio cue alone and either remain within or correctly return to the inclusion zone when stimuli were applied. The sheep were left in the paddock with the virtual fence line until the trial concluded on the morning of day five. However, the fence was temporarily deactivated when all animals were brought into the yards briefly each morning to check the correct positioning of the units and that there was no rubbing or abrasions caused by the harness, as well as to fix any wiring that had become loose. Time in the yards ranged from 43–60 min and was excluded from any data analyses. Animals were regularly visually checked in person in the paddock across the five-day trial period as well as on the online user interface.

### 2.3. Lameroo Commercial Farm Trial—Animals and Experimental Protocol

Following the five-day pilot trial in Armidale, NSW, a small trial was conducted on a commercial farm in Lameroo, SA, during October 2022. The test paddock was a mix of weeds and barley grass, with plentiful grazing feed available and a single water point within the inclusion zone. On day one (morning), the same modified eShepherd^®^ devices were fitted in a race to a group of 10 Ultra White ewes naïve to virtual fencing, with necks clipped and numbers sprayed on their wool. The animals were approximately 8 months of age and estimated to be an average of 60 kg, based on weights of the larger farm flock taken close to the commencement of the trial. As per the Chiswick trial, animals were given minimum 30 min to acclimate to the device. The sheep were then walked 215 m to their 1.3 ha test paddock, where a virtual line had already been set across the width of the paddock, excluding 47% of the area. As part of the training period, personnel initially stood in the exclusion zone to prevent the sheep running through the virtual line, as the entry gate for the animals was at the top of the inclusion zone. However, all animals slowly walked into the upper inclusion zone and began grazing; thus, personnel left the paddock. Researchers remained in an adjacent paddock observing the animals throughout the day. The sheep all crossed into the exclusion zone during their first interactions, as they were learning the cues. After an approximately 1-h training period, seven sheep had correctly returned to the inclusion zone. After approximately 90 min in total, three sheep that remained within the exclusion zone were walked up to the inclusion zone by personnel. This was considered the end of the training period and thus the end of personnel intervention. In previous cattle trials with the eShepherd^®^ devices, personnel did not intervene, and any ‘training period’ was defined by the length of time until all animals remained in the inclusion zone and were responding to the audio cue alone [9,10,11]. The signals emitted by the devices are always the same, regardless of level of naivety of the animals, but cattle may need more ‘training’ to move from a simple straight virtual line to a more complex contoured virtual line [11]. Personnel intervened in the current trial to minimise any welfare impacts on the animals that were not showing understanding of the system and remaining in the exclusion zone. This was not observed in previous cattle trials [9,10,11]. Once the animals were all back together, they remained in the test paddock until the following morning’s check. All animals were brought back into the yards each morning to check the fit of the devices and repair any connecting wires that had broken. Time in the holding yards ranged from 50–100 min and was excluded from any data analyses. Animals were visually checked in person periodically each day as well as on the online user interface. The trial concluded on the morning of day five, and all devices were removed.

### 2.4. Data

Data from the neckband devices were extracted per individual animal (Chiswick: n = 6; Lameroo: n = 10) from a cloud database and included the cumulative total number of audio cues and electrical pulses each animal received across the trial duration and sampled GPS location readings every 10 min (latitude and longitude), with an accuracy of approximately ±5 m. Any GPS data recorded when animals were yarded were removed from the datasets. GPS data were also filtered to within a 10 m buffer surrounding the outside of the test paddock to remove recordings that were affected by GPS drift (46 datapoints of 3078 were removed for Chiswick; 307 datapoints of 6769 were removed for Lameroo). The devices, while operating on the animal during the trials, monitored each animal’s GPS location more frequently to be able to accurately deliver signals when appropriate. However, the data recorded directly to the cloud only saved locations every 10 min, as this was the sampling period. The total time spent in the exclusion zone was calculated by counting all GPS records and multiplying by 10 min, by 60 s, then dividing by the total number of seconds in the trial (minus the yarded time). For Chiswick, the total audio cue and electrical pulse data per animal per day were used to calculate the average number of audio cues and electrical pulses received across the trial period across all animals as well as the mean percentage of total administered audio cues that were audio only (i.e., not followed by an electrical pulse; total audio cues minus electrical pulses/total audio cues) * 100). For the Lameroo trial, these audio cue and electrical pulse calculations were separated into the training period as well as daily averages for the five-day trial and for the incursion period when the animals broke through the boundary, until they were walked out the following morning (01:00–09:00). Daily audio cue and electrical pulse totals are visually presented per individual sheep per day. For Chiswick, the 10-min GPS location data were used to plot the locations of individual animals throughout each day across the trial period. For Lameroo, the 10-min GPS location data were used to plot the locations of individual animals throughout each day across the trial period, as well as hourly locations on the first and fourth day of the trial. All GPS data were plotted in R statistical software [22] using the package ‘ggplot2′ [23].

## 3. Results

**Figure 2:**
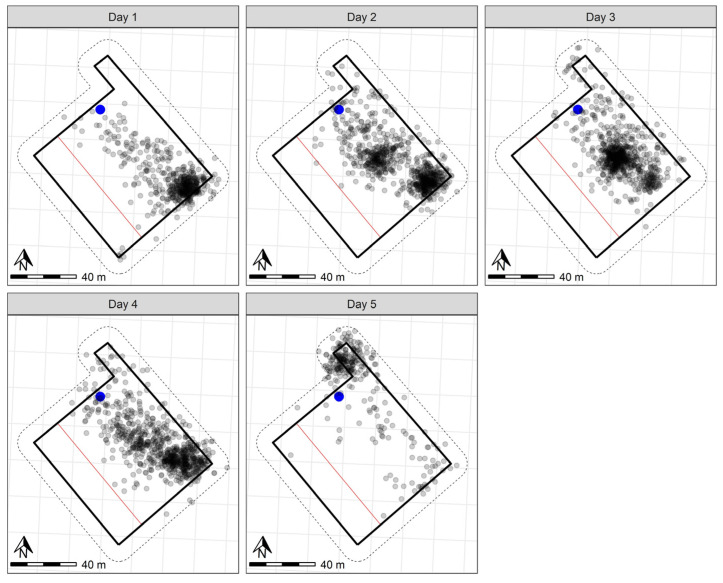
GPS plots per day for six sheep in the five-day Chiswick trial with the water point indicated in blue. The grey points indicate the GPS positions (measured every 10 min) of individual sheep throughout the day. Darker points indicate point overlay in locations where sheep were registered more frequently. The solid black outline indicates the wire fenced paddock boundary. The dashed lines on the outside of the paddock boundary indicate the 10 m buffer zone used to clip the data to remove recording errors based on GPS drift. The red line across the width represents the virtual fence. A scale is included to indicate paddock size.

The six sheep at Chiswick were contained within the inclusion zone for 99.8% of the trial period (Figure 2). Primarily, the incursions into the exclusion zone were on the first day during learning (Figure 2). Across the entire trial period, the mean audio-only percentage (±SD) was 74.93% ± 4.6%. Across all individuals, the mean number (±SD) of audio cues received was 7.8 ± 8.3, and the mean number (±SD) of electrical pulses received was 1.6 ± 2.2.

**Figure 3:**
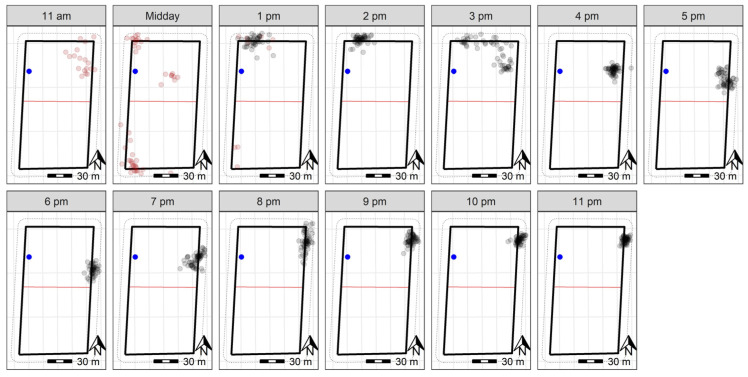
GPS plots across 11 h on day one of the trial for 10 sheep at Lameroo, with the training period indicated in red datapoints and the water point indicated in blue. The grey points (or red during training) indicate the GPS positions (measured every 10 min) of individual sheep throughout the day. Darker points indicate point overlay in locations where sheep were registered more frequently. The solid black rectangle indicates the wire fenced paddock boundary, with the red line representing the virtual fence. The dashed lines on the outside of the paddock boundary indicate the 10 m buffer zone used to clip the data to remove recording errors based on GPS drift. A scale is included to indicate paddock size.

**Figure 4:**
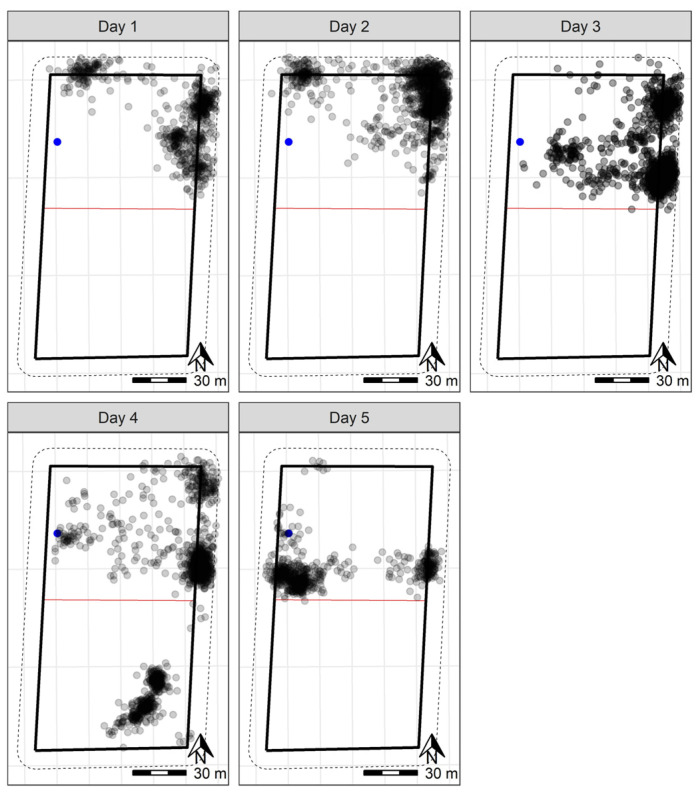
GPS plots per day for 10 sheep at Lameroo, with the water point within the inclusion zone indicated in blue. The grey points indicate the GPS positions (measured every 10 min) of individual sheep throughout the day. Darker points indicate point overlay in locations where sheep were registered more frequently. The solid black rectangle indicates the wire fenced paddock boundary, with the red line representing the virtual fence. The dashed lines on the outside of the paddock boundary indicate the 10 m buffer zone used to clip the data to remove recording errors based on GPS drift. These plots do not include the initial training period on day one that is displayed in Figure 3. A scale is included to indicate paddock size.

**Figure 5:**
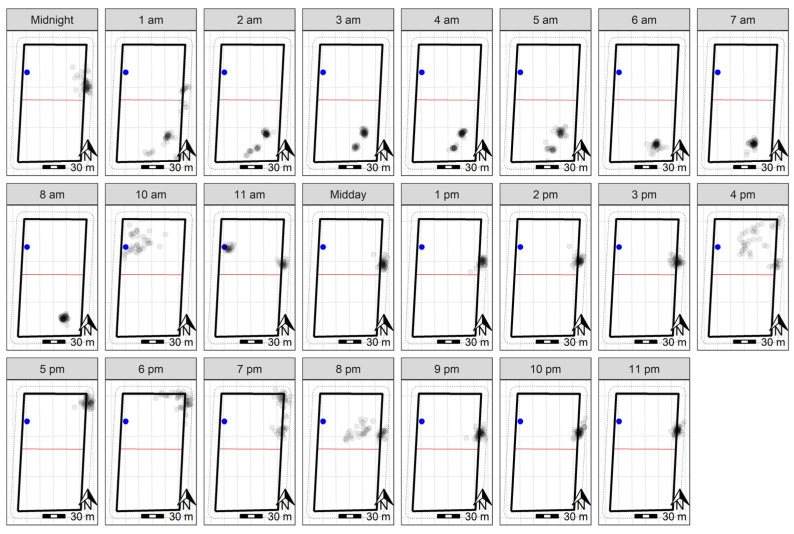
GPS plots across 23 h on day four of the trial for 10 sheep at Lameroo, with the water point within the inclusion zone indicated in blue. The grey points indicate the GPS positions (measured every 10 min) of individual sheep throughout the day. Darker points indicate point overlay in locations where sheep were registered more frequently. The solid black rectangle indicates the wire fenced paddock boundary, with the red line representing the virtual fence. The dashed lines on the outside of the paddock boundary indicate the 10 m buffer zone used to clip the data to remove recording errors based on GPS drift. The data at ‘9 am’ were removed, as the animals were yarded at this time. A scale is included to indicate paddock size.

**Figure A1:**
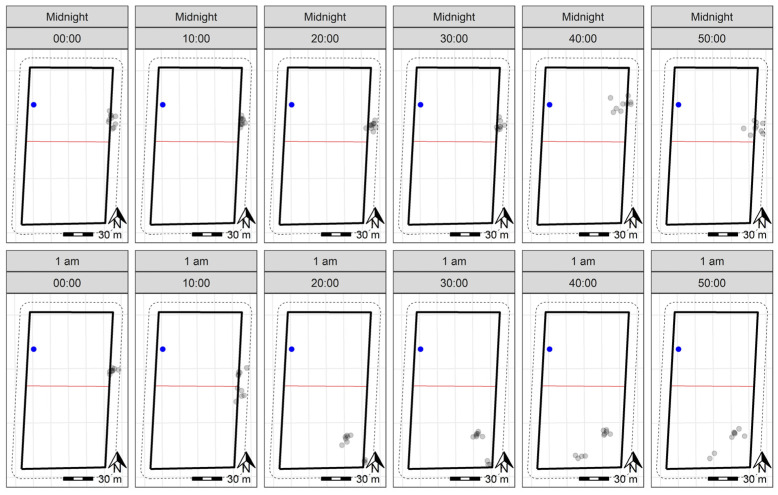
GPS plots across 10 min periods from midnight until 02:00 on day four of the trial for 10 sheep at Lameroo, with the water point indicated in blue. This represents the incursion period when the animals crossed through into the exclusion zone and remained there overnight. The grey points indicate the GPS positions (measured every 10 min) of individual sheep throughout the day. The solid black rectangle indicates the wire fenced paddock boundary, with the red line across the width representing the virtual fence. The dashed lines on the outside of the paddock boundary indicate the 10 m buffer zone used to clip the data to remove recording errors based on GPS drift. A scale is included to indicate paddock size.

Following the 90-min training period, the 10 sheep at Lameroo were contained within the inclusion zone for 92.2% of the trial period (Figure 3 and Figure 4). They did cross over into the exclusion zone on the third night at approximately 01:00. Downloaded GPS logs indicated all animals moved from the inclusion to exclusion zone within a 10-min window (Figure A1), but observation of animal movement in the user interface the following morning indicated it was a rapid flock movement within a few minutes. The online user-interface enabled more precise tracking of the animals’ movements, as additional data were transmitted when an animal interacted with the fence line. However, the data delivery system had limitations in the amount of data it could send; thus, the downloaded cloud logs were the primary datasets used. The animals remained in the exclusion zone until they were walked out by personnel for their daily yard check around 09:00 the morning of the fourth day (Figure 5). During the time in the exclusion zone, the animals moved around in one corner of the paddock initially, then settled as two groups in proximity, before combining as one group (Figure 5) until the morning. Once the animals were walked back to the inclusion zone following their yard check, they correctly remained in the inclusion zone until the conclusion of the trial (Figure 4 and Figure 5).

**Figure 6:**
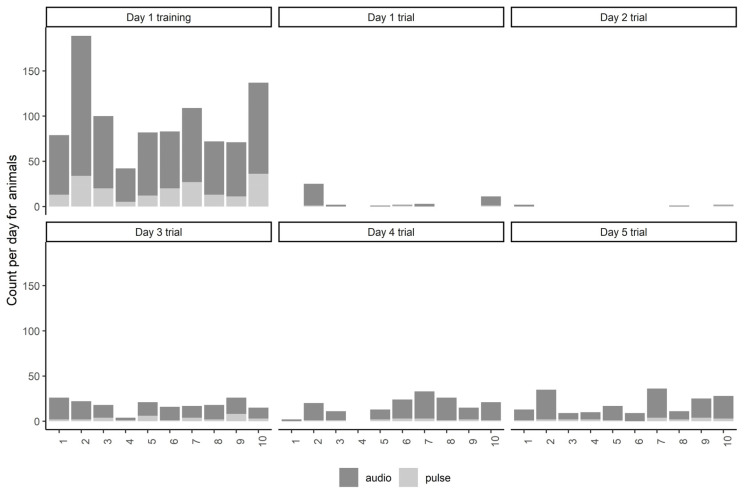
The total number of audio cues and electrical pulses per individual animal across each day of the trial at Lameroo, including during the initial training period on day 1. The incursion period where animals crossed into the exclusion zone on the third night (01:00–09:00) is not displayed (mean data for this period are presented in Table 1).

During the 90-min training period before personnel intervened, the mean percentage of total audio cues that were audio only (±SD) was 76.2 ± 7.4%, during the incursion period on the third night it was 87.4 ± 6.7%, and for the remainder of the trial it was 82.2 ± 22.9%. Across all individuals, the mean number (±SD) of audio cues received during the training period was 77.3 ± 32.1, and the mean number (±SD) of electrical pulses received during training was 19.1 ± 10.3. For the remainder of the trial period (including the incursion event), the mean number (±SD) of audio cues received was 31.4 ± 57.2, and the mean number (±SD) of electrical pulses received was 4.2 ± 7.9. The mean daily audio cue and electrical pulse values are presented in Table 1, including the training period and the incursion period, when the animals crossed over into the exclusion zone. The highest numbers of stimuli were received during training and the incursion period (Table 1). Daily audio cues and electrical pulses per individual animal are displayed in Figure 6, which indicates high individual variation in received stimuli and thus interactions with the fence line.

## 4. Discussion

This study applied modified eShepherd^®^ virtual fencing neckbands to sheep using harnessed units wired to collars to enable automated trials with two small groups of sheep across both the day and night. The results showed the sheep were able to appropriately respond to the virtual fencing cues and learn to respond to the audio cue alone with similar audio-only percentages to cattle wearing the eShepherd^®^ devices [9,11]. The animals were predominantly kept in the inclusion zone across the five-day trial periods, but there was one instance where sheep rapidly broke through the boundary at night, possibly due to an animal scare, and remained within the exclusion zone for approximately 8 h until they were walked back to the yards by personnel. It is unknown when they would have returned to the inclusion zone without personnel intervention. The results of these preliminary trials are promising for the use of automated technology on sheep, but suitable devices still need to be designed that can be applied longer-term, with algorithms specific to sheep behaviour that will successfully herd them back into the inclusion zone. The necks of these reduced-wool sheep were clipped to ensure consistency of contact; thus, a collar device would need sufficient electrode contact and may only be applicable to some sheep breeds. A device that uses aversive audio cues may suit multiple sheep breeds, but habituation to the signals longer-term is probable, as sheep would likely learn to ignore the cue if there are no meaningful consequences. Alternatively, an ear tag could be viable if the required components can be reduced to a small-enough weight.

The relatively successful results of the two automated trials were similar to what has been previously shown using manually operated dog collars on sheep [14,15,16]. Sheep predominantly remained in the inclusion zone and showed responses to the audio cues alone, with variation between individuals in learning rate as well as fence interaction frequency. As described by Lee and Campbell, 2021 [24], the inclusion of a metric such as the relative proportions of audio cues and electrical pulses is informative with regard to the animal welfare impacts of virtual fencing. This metric indicates if animals are learning to avoid the electrical pulse and respond to the audio cue alone [25], which has been shown to minimise stress responses to virtual fencing cues in sheep [26]. However, the high individual variation indicates some sheep may have been experiencing different degrees of welfare impact during the learning process. The audio-only cue percentages were similar to what has been observed in previous cattle trials using the eShepherd^®^ devices ([9]: 71.5%; [11]: 74.5%). Early work applying the Nofence automated devices concluded that virtual fencing may not be suitable for sheep due to limited containment success and welfare concerns [7,8]. In the first trials with Nofence, only 37.5% of the test ewes reached the pre-established learning criterion of responding to the audio cue alone after three repetitions of the audio-pulse sequence [7]. However, this is a strict learning criterion, designed to minimise negative impacts of the electrical pulses on animal welfare. Previous work with manual collars has shown 22% of sheep responded to the audio cue alone after their third interaction and 67% of sheep did so by the fifth interaction [14], similar to early studies with cattle, where 56% of animals correctly responded by the fifth interaction [27]. These calculations could not be made in the current study with the way the device data were recorded, but all animals showed responses to the audio cue alone within the approximate 90-min training period.

Trials also ceased in [8], as the majority of sheep exceeded the pre-set criterion of a maximum of four shocks on the second day of the trial. Mean electrical pulse values in the current study far surpassed the criterion in [8] during the training period and when the group of animals broke through into the exclusion zone. However, it is unclear if every delivered pulse in the current study was aversively felt by the individual. In the study in [7] using Norwegian white and Spæl sheep, many individuals were removed because of severe running reactions, but conversely, other individuals were removed due to no visible reaction to the pulse. In a follow-up study with the same breeds, similar individual variation in sensitivity to the same pulse strength was observed; however, animals in the test groups were not preselected [8]. This variation in sensitivity to the electrical pulse could hinder learning for the individual receiving the electrical pulses as well as for other individuals within the flock through social facilitation. Partial records of electrical pulse delivery in the current trial indicated some electrical pulses were likely not of sufficient contact to be felt, but these datasets were not complete given the limitations in data transfer capacities. The studies with the Nofence devices used earlier prototypes that had several technical issues; this could have contributed to the poor learning outcomes. More recent trials have successfully applied Nofence devices to cattle for an extended period of time [1] and to small goat groups for five days [6]. If animals are receiving a high number of aversive electrical pulses, then this would need to be addressed before an automated system can be considered a welfare-friendly fencing alternative. A managed training period on-farm could mitigate some of the risks with this individual variation. However, individual variation in sensitivity, which could be exacerbated by variation in wool coverage and/or patchy wool shedding, could increase the inconsistency in electrode contact and animal responses in the long term.

The current trials were the first to apply the eShepherd^®^ algorithm by leaving devices on to run continuously across the day and night in sheep (minus daily yard checks), confirming the on-farm feasibility for this technology. However, there was an instance during the Lameroo trial where the flock crossed over the boundary at night and remained within the exclusion zone until the following morning. The eShepherd^®^ system operates so that animals still receive audio cues and electrical pulses if moving farther into the exclusion zone (but not if moving in the direction out of the exclusion zone), unless they reach a predetermined limit and initiate a temporary cue timeout. There are no different cues emitted to communicate the breach to the animal. The animals spent the time in one corner of the exclusion zone, predominantly stationary, presumably lying down. It is unclear precisely what led to the sheep running through the boundary. Regardless of the reason, the system is designed to herd cattle back in such situations, so it is also unclear if the sheep did not know how to return, or if the location was preferred for nighttime resting. Sheep are highly gregarious, with some individuals and even breeds more gregarious than others [28,29]. Furthermore, social isolation is a significant stressor for sheep [30]. This could present issues if there are one or two individuals that may cross through the boundary and attract others to follow. Similarly, once the sheep are in the exclusion zone, the automated algorithm may struggle to herd them back if it is working against the flocking instinct. Cattle trials have shown the virtual fence is less effective when there is close visual contact with other cattle groups across a boundary [31]. Trials with automated dog collars using a buried wire system showed that if naive sheep crossed the boundary, their trained peers would occasionally follow after them [12]. Similar issues were observed when only some animals had functioning Nofence collars [8]. When investigating whether controlling only some sheep within a flock with virtual fencing devices was effective at containing sheep, Marini et al. (2020) [18] showed that a minimum of 66% of sheep needed to be virtually fenced to contain the whole group. It should be noted that this was for a short period with only nine animals; longer time frames and larger animal numbers may reduce the effectiveness. Conflict between the aversive electrical pulses and a desire to reunite with conspecifics have also been demonstrated in cattle [19]. Thus, there needs to be further work to understand how sheep may be encouraged to return to the inclusion zone in situations where the entire group has crossed over. This further work would also need to extend to fencing situations where there are multiple virtual boundaries (i.e., a virtual enclosure), rather than a single virtual boundary combined with physical fences. Algorithms that are specific to sheep behaviour may need to be developed for the automated technology to operate successfully. More precise GPS movement detection may accommodate the smaller and potentially more rapid direction changes of sheep. In the current study, it is unknown if the sheep would have eventually returned if they had not been walked back by personnel for their yard checks. Similarly, personnel intervened at the conclusion of the training period at Lameroo to minimise any welfare compromises to the few sheep that were separated from the group and remained within the exclusion zone.

## 5. Conclusions

These preliminary trials with automated devices indicate there is potential for virtual fencing technology to be successfully applied to sheep, but suitable devices still need to be validated for longer-term work. A harness should only be applied in the short term due to the potential for rubbing, and any internal algorithms based on the animal’s behaviour need to be developed and validated specifically for sheep rather than cattle. Demonstration that all sheep in a group can be successfully herded back if they do break through into the exclusion zone is still required. Devices for sheep are limited by the challenges of smaller body weight and wool. Thus, electrical pulses administered via collars may only work for some breeds with reduced wool covering if sufficient electrode contact is able to be sustained. While longer-term validation of automated devices is required to assess their functionality and animal impacts, there could be value in shorter-term on-farm applications of the technology. Targeted grazing for shorter periods with small numbers of animals, such as in weedy areas within paddocks, could be made possible by this technology, opening up new management opportunities for producers.

## Figures and Tables

**Table 1:** Daily mean (±SD) number of audio cues and electrical pulses across all 10 animals at Lameroo ^1^.

Date	Period	Mean Audio ± SD	Mean Pulse ± SD
Day 1	Training	77.3 ± 32.1	19.1 ± 10.3
Day 1	Trial	4.1 ± 7.6	0.3 ± 0.5
Day 2	Trial	0.4 ± 0.7	0.1 ± 0.3
Day 3	Trial	15 ± 5.5	3.3 ± 2.3
Day 4	Trial	15.1 ± 9.7	1.4 ± 1.1
Day 4	Incursion	136.7 ± 78.3	17.9 ± 11.9
Day 5	Trial	17.2 ± 10.0	2.1 ± 1.3

^1^ The training period was 90 min in duration. Incursion refers to the instance where the animals broke through the fence overnight (01:00) and remained in the paddock until they were walked out by personnel the following morning (09:00).

## Data Availability

Data supporting this study are available upon request to the corresponding authors.
